# Factors Affecting Cecal Intubation Time in Colonoscopy: Impact of Obesity

**DOI:** 10.7759/cureus.15356

**Published:** 2021-05-31

**Authors:** Beslen Goksoy, Mevlut Kiyak, Mehmet Karadag, Gokhan Yilmaz, Ibrahim F Azamat

**Affiliations:** 1 General Surgery, Sehit Prof. Dr. Ilhan Varank Sancaktepe Training and Research Hospital, University of Health Sciences, İstanbul, TUR; 2 Gastroenterology, Sehit Prof. Dr. Ilhan Varank Sancaktepe Training and Research Hospital, University of Health Sciences, Istanbul, TUR; 3 Biostatistics, School of Medicine, University of Hatay Mustafa Kemal, Hatay, TUR; 4 General Surgery, Istanbul Medipol University, Istanbul, TUR; 5 General Surgery, Istanbul University Istanbul Medical Faculty, Istanbul, TUR

**Keywords:** cecal intubation time, colonoscopy, obesity, waist-to-height ratio, waist circumference

## Abstract

Objective

This study aims to determine the factors that prolong cecal intubation time (CIT) and determine the effect of obesity on CIT measured using multiple indexes.

Methods

Patients who underwent elective colonoscopy between July 10, 2020, and January 20, 2021, were evaluated in this prospective observational study. Age, gender, constipation, bowel preparation, presence of diverticulosis, previous surgery history, auxiliary maneuver and additional analgesic requirement, cecum intubation length (CL) and obesity indices [body mass index (BMI), waist circumference (WC), waist-to-height ratio (WHtR)] were analyzed. Factors affecting CIT were assessed by both univariate and multivariate logistic regression (LR) analyses.

Results

A total of 512 patients were analyzed. Mean CIT was 5.6 ± 1.6 min, and median CIT was 5.17 min. The CIT median was ≤5.17 min in 264 (51.5%) of the patients, and the CIT median was >5.17 min in 248 (48.5%). In the univariate LR results, young age, constipation, poor bowel preparation, increased CL, additional analgesic requirement, low WHtR, and low BMI (<25 kg/m2) were the factors that prolonged CIT (p <0.05). In the multivariate LR analysis results, WHtR >0.5 and BMI >30 kg/m2 were found to be independent factors that decrease CIT [OR: 0.01 (0.01 0.03) p <0.001; OR: 0.28 (0.13 0.57) p <0.001].

Conclusion

Younger age, low WHtR, low BMI, increased CL, constipation, inadequate bowel preparation, and the use of extra analgesics were found to be associated with longer CIT. When all factors were evaluated together, obesity measured by only WHtR (>0.5) and BMI (>30 kg/m2) were the best predictors of decreased CIT.

## Introduction

Colonoscopy is considered to be the primary screening test for colorectal cancer screening worldwide [1]. Prolongation of cecal intubation time (CIT), which is one of the quality indicators in colonoscopies, decreases adenoma detection rate, causes delay in diagnosis and treatment, and negatively affects patient comfort [2,3].

In a systematic review and meta-analysis conducted in 2018, factors affecting CIT were examined, and it was reported that elderly patients, female gender, low body mass index (BMI), and poor bowel preparation prolonged CIT [4]. On the other hand, previous abdominal surgery and constipation have been shown in some studies to prolong CIT, but other studies have stated that they don’t affect CIT [4-6]. Hence, the results of these previous studies are contradictory.

In a limited number of studies, the effects of obesity indices such as BMI, waist circumference, and visceral and subcutaneous adipose tissue on CIT have been investigated, and CIT is shorter in overweight patients [7-9]. Although BMI is the most commonly used obesity index, it does not differentiate muscle from fat and fails to predict body fat [10]. Waist-to-length ratio (WHtR) is a simple measurement method such as WC and BMI. A recent study showed that WHtR is more predictive than BMI in estimating visceral fat ratio [11].

This study aims to determine the factors that prolong CIT and determine the effect of obesity indexes measured by BMI, WC, and WHtR on CIT.

## Materials and methods

Study design and population

This prospective observational study was carried out in a center where more than 10,000 endoscopic procedures are performed annually and where tertiary healthcare services can be performed simultaneously with multiple endoscopic procedures. Patients who underwent an outpatient elective colonoscopy procedure between July 10, 2020, and January 20, 2021, were evaluated. Inclusion criteria were patients older than 18 years, patients who performed bowel preparation given before the procedure, and patients with the American Society of Anesthesiologists Class (ASA) I-III. Patients with anesthesia or analgesic allergy, patients undergoing multiple procedures at the same time, pregnant patients, patients with a history of colorectal surgery, patients with active inflammatory bowel disease, emergency procedures, patients with liver cirrhosis or ascites, and patients in whom the cecum could not be reached were excluded from the study. A written informed consent form was obtained from all patients before the procedure. All procedures followed to comply with the committee's ethical standards (institutional and national) responsible for human experimentation and the 1964 Declaration of Helsinki and later versions. This study was approved by the Local Ethics Committee (protocol number: 074, date: 30.04.2020). All authors reviewed the study data and reviewed and approved the final manuscript.

Procedure

Four liters of polyethylene glycol-electrolyte solution (GoLYTELY®; Avicenna Farma, Istanbul, Turkey) was used for bowel preparation in all patients. All patients were placed in the lateral decubitus position, and the colonoscopy procedure was started. All procedures were performed under sedation (midazolam + pethidine hydrochloride) adjusted for the patient's weight with a high-resolution video endoscopy system (VP-4450HD; Fujifilm, Tokyo, Japan) and an adult colonoscopy device (EC-530 series; Fujifilm). When necessary, additional narcotic analgesic was administered and recorded. The visualisation of the appendix orifice and the ileocecal valve was defined as the indication of cecal intubation and was documented in the procedure report and photographed. When the cecum was reached, the colonoscope length (CL) was recorded in centimeters. Diagnosis and treatment attempts such as polypectomy or biopsy related to pathologies were performed during withdrawal after the cecum was reached and did not affect CIT. Auxiliary maneuver (manual pressure and position change) requirement was applied by experienced assistant personnel at the discretion of the endoscopist. The quality of bowel preparation was evaluated according to the Boston Bowel Preparation Scale and was divided into two groups for analysis: 1) excellent-good, 2) moderate-poor [12]. All procedures were performed by four endoscopists with more than 1500 colonoscopy experience.

Exposure variables

Age, gender, height (cm), weight (kg), and waist circumference (cm) (WC) of all participants were recorded before colonoscopy. They were also asked to fill out a questionnaire stating whether they had comorbidity, constipation, and previous abdominal surgery history. The auxiliary maneuvers performed during colonoscopy and the use of extra analgesics, if any, were recorded. After colonoscopy, the quality of bowel preparation, CIT, colonoscope length (CL), and diverticulosis presence were recorded. A 10-point visual analog scale (VAS) was used to assess post-procedure pain and was recorded before discharge.

Obesity indices measurement

WC was measured in the horizontal plane between the lowest ribs and the iliac crest. BMI was calculated by dividing the weight by the square of the height (kg/m2). WHtR was calculated as WC (cm)/height (cm). The patients were divided into groups in terms of all three obesity indices: 1) normal BMI 18.5-24.9 kg/m2, overweight BMI 25-29.9 kg/m2, obese BMI ≥30 kg/m2, 2) normal WC (for men <100 cm, for women <90 cm) and high WC (for men 100 cm, for women 90 cm), 3) normal WHtR <0.5 and high WHtR ≥0.5 [13].

Statistical analysis

The suitability of the data to normal distribution was evaluated with the Kolmogorov-Smirnov test. Comparison of normally distributed features in two independent groups Student's t-test was used, and the Mann Whitney U test was used to compare non-normally distributed features in two independent groups. Relationship analysis of categorical variables was analyzed using Exact and Pearson Chi-square tests. The CIT level's median value was considered the cut-off value for the duration of the procedure and divided into two groups: group 1 ≤ CIT median value and group 2 > CIT median value. In addition to some clinical characteristics, age, BMI, laboratory, and treatment methods were analyzed first with the Univariate LR (Logistic Regression) method. Then, the effects of variables found to be significant (P <0.05) on the CIT time using the Stepwise Multivariate Enter LR method. Median and quarters for numerical variables and number and % values ​​for categorical variables are given as descriptive statistics. SPSS Windows version 23.0 (IBM Corp., Armonk, NY, USA) was used for statistical analysis, and p <0.05 was considered statistically significant.

## Results

Baseline characteristics

During the study period, a total of 904 patients underwent colonoscopy. Three hundred eleven patients who did not meet the initial study criteria (multiple simultaneous procedures n=142, emergency procedures n=49, history of colorectal surgery n=38, not performing bowel preparation n=30, inflammatory bowel disease n=28, possible pregnancy n=8, intraabdominal ascites n=8, possible allergy n=8) were excluded. After 81 of the remaining 593 patients were excluded from the study for various reasons (missing procedure n=32, screen failure n=25, lack of clinical data n=24), a total of 512 patients were included in the study. The mean age was 51.1 ± 11.8 years, and 56.3% (n=288) were women. The mean CIT time was 5.6 ± 1.6 min, and the median CIT time was 5.17 min (range 1.6-14.4). The mean BMI of the participants was 26.2 ± 4.9 kg/m2, WC 93.08 ± 13.82 cm, and WHtR was calculated as 0.49 ± 0.09. General characteristics and colonoscopy findings of the participants are given in Table 1.

**Table 1:** Distribution of general characteristics and colonoscopy findings BMI: body mass index; VAS: visual analog scale; ASA: American Society of Anesthesiologists

	Median	Mean±SD	Min-Max
Age	50.00	51.16±11.82	19-84
BMI	26.85	26.22±4.91	16-55
Waist circumference (cm)	92.00	93.08±13.82	65-138
VAS score	3.00	2.92±1.78	0-8
Cecal intubation time (min)	5.17	5.65±1.64	1.67-14.42
Cecal intubation length (cm)	95.00	95.77±17.90	50-160
Waist-to-height ratio	0.48	0.49±0.09	0.4-0.9
	n (%)		
Gender			
Male	224 (43.8)		
Female	288 (56.3)		
Waist-to-height ratio			
≤0.5	282 (55.1)		
>0.5	230 (44.9)		
Constipation			
Yes	62 (12.1)		
No	450 (87.9)		
Bowel preparation			
Good	458 (89.5)		
Bad	54 (10.5)		
Previous surgery			
Yes	54 (10.5)		
No	458 (89.5)		
Auxiliary maneuver			
Yes	146 (28.5)		
No	366 (71.5)		
Diverticulosis			
Yes	38 (7.4)		
No	474 (92.6)		
Extra analgesic requirement			
Yes	46 (9.0)		
No	466 (91.0)		
ASA			
1	398 (77.7)		
2	90 (17.6)		
3	24 (4.7)		

Comparison of demographic and clinical characteristics according to the CIT median value

The CIT median was ≤5.17 min in 264 (51.5%) of the patients, and the CIT median was >5.17 min in 248 (48.5%). Comparing the two groups according to the median value of the CIT, age (p = 0.025), CL (p = 0.001), bowel preparation (p = 0.011), BMI (p <0.001), WHtR (p <0.001), constipation (p <0.001) and the use of extra analgesic (p = 0.003) were found to be statistically significantly different (Table 2).

**Table 2:** Demographic and Clinical Characteristics According to the CIT CL: cecum intubation length; BMI: body mass index; WC: waist circumference; WHtR: waist-to-height ratio; ASA: American Society of Anesthesiologists; M: male; F: female P values were obtained from chi-square test for categorical variables and Mann Whitney U test for quantitative variables. (IQR: Inter Quartile Range=Q3-Q1)

		CIT				
		≤5.17 (n=264)		>5.17 (n=248)		p
Gender n,%	Male	119	45.1	105	42.3	0.538
	Female	145	54.9	143	57.7	
Age median (IQR)		52 (17)		49 (15)		0.025
CL (cm) median (IQR)		90 (22)		96 (20)		0.001
		n, %		n, %		
BMI	18.5-24.9	73	27.6	100	40.3	<0.001
	25-29.9	110	41.7	108	43.6	
	>30	81	30.7	40	16.1	
WC	M<100 & F<90	112	42.4	120	48.9	0.052
	M≥100 & F≥90	152	57.6	128	51.6	
WHtR	≤0.5	118	44.7	164	66.1	<0.001
	>0.5	146	55.3	84	33.9	
Constipation	Yes	19	7.2	43	17.3	<0.001
	No	245	92.8	205	82.7	
Bowel prep	Good	245	92.8	213	85.9	0.011
	Bad	19	7.2	35	14.1	
Previous surgery	Yes	22	8.3	32	12.9	0.092
	No	242	91.7	216	87.1	
Auxiliary maneuver	Yes	83	31.4	63	25.4	0.131
	No	181	68.6	185	74.6	
Diverticulosis	Yes	17	6.4	21	8.5	0.766
	No	247	93.6	227	91.5	
Extra analgesic requirement	Yes	14	5.3	32	12.9	0.003
	No	250	94.7	216	87.1	
ASA	1	198	75.0	200	80.6	0.111
	2	49	18.6	41	16.5	
	3	17	6.4	7	2.8	

The factors thought to have an effect on the CIT level being higher than the median = 5.17 were first examined by univariate logistic regression (LR), then the variables that were observed to be statistically significant (p <0.05) in the multivariate LR model, and the contribution of variables was examined using the enter method. Univariate LR results showed that young age, low WHtR, constipation, poor bowel preparation, additional analgesics, and low BMI (<25 kg/m2) alone significantly prolonged CIT. In the multivariate LR analysis, WHtR >0.5 and BMI >30 kg/m2 were found to be independent factors associated with decreased CIT [OR: 0.01 (0.01 0.03) p <0.001; OR: 0, 28 (0.13 0.57) p <0.001] (Table 3).

**Table 3:** Univariate and Stepwise Multivariate LR Analysis of Prediction of CIT Level CL: cecum intubation length; WHtR: waist-to-height ratio; ASA: American Society of Anesthesiologists; BMI: body mass index Odds Ratio values were obtained from Logistic regression models. Final Model Nagelkerle R2=0.54

	Univariate LR		Stepwise LR	
	Odds Ratio (95% CI )	p	Odds Ratio (95% CI )	p
Age	0.98 (0.97 0.99)	0.020	1.00 (0.98 1.02)	0.991
Male	1.12 (0.79 1.59)	0.533		
CL	1.02 (1.01 1.03)	0.001	0.99 (0.98 1.01)	0.377
WHtR >0.5	0.01 (0.01 0.02)	<0.001	0.01 (0.01 0.03)	<0.001
Constipation	2.71 (1.53 4.79)	<0.001	1.81 (0.84 3.89)	0.130
Poor bowel preparation	2.12 (1.18 3.81)	0.012	1.67 (0.77 3.63)	0.191
Previous surgery	0.61 (0.35 1.09)	0.095		
Auxiliary maneuver	0.74 (0.51 1.09)	0.131		
Diverticulosis	1.34 (0.69 2.61)	0.383		
Extra analgesic	2.65 (1.38 5.08)	0.004	1.35 (0.54 3.38)	0.518
BMI (ref: 18.5-24.9)		<0.001		<0.001
25-29.9	0.31 (0.18 0.53)	<0.001	0.65 (0.32 1.29)	0.217
>30	0.17 (0.10 0.30)	<0.001	0.28 (0.13 0.57)	0.001
ASA (ref:1)		0.123		
2	0.83 (0.52 1.31)	0.421		
3	0.41 (0.17 1.01)	0.051		

## Discussion

In this study, young age, increased CL, low WHtR, low BMI, constipation, additional analgesic requirement, and insufficient bowel preparation were associated with longer CIT. On the other hand, increased WHtR and BMI were independent predictors of decreased CIT.

In previous studies, it has been reported that excess weight measured by BMI shortens CIT [4,14-16]. There are several possible causes for this situation. First, in overweight patients, more intra-abdominal fat supports the colon and provides fewer folds during colonoscopy [7,9]. Second, obese patients have a relatively shorter colon [17]. Third, the angulation in thin patients causes more pain, which causes prolongation of the procedure time. On the other hand, no relationship was found between BMI and CIT in the study performed by Hsu et al. The authors attributed this to the fact that the pain factor was excluded because the procedures were performed under deep sedation [6]. Nagata et al. compared the fat ratio measured by computed tomography (CT) in addition to BMI for obesity index and reported that subcutaneous fat accumulation measured by CT was the best determining factor for easier colonoscopy [7]. Although it allows more specific determination of body fat ratio, CT application before colonoscopy does not seem practical in cost and patient safety. In another study, Hsieh et al. stated that WC is a better predictor than BMI in evaluating CIT [15]. It has been reported that WHtR is more predictive than BMI and especially WC in male patients in estimating visceral fat ratio [11]. Based on this, we used WHtR in addition to BMI and WC for obesity index in our study. Similar to the literature, shorter CIT was obtained in overweight patients in our study. When the obesity indexes were compared, BMI and WHtR were significant in evaluating CIT (p <0.001). WC showed close significance but was not statistically significant (p = 0.052). In univariate LR analysis, BMI >25 kg/m2 and WHtR >0.5 shortened CIT, whereas in multivariate LR analysis results, only BMI >30 kg/m2 and WHtR >0.5 shortened CIT.

The relationship between age and CIT has been examined in some studies, and conflicting results have been reported [4,7,16]. The increase in colon length and elasticity with aging may cause prolongation of CIT [17]. In our study, unlike the literature, we found a longer CIT in younger patients. Young people may experience more pain during colonoscopy due to the tighter mesenteric structure. This may cause CIT prolongation by requiring additional analgesics and making it difficult for the colonoscopist to focus on the procedure [18]. However, this relationship is unclear and needs to be studied further.

The relationship between bowel preparation, one of the quality indicators in colonoscopy, and the CIT is not clear [4,19,20]. In our study, we found that better bowel preparation reduces CIT. This is not surprising because poor bowel preparation causes more time to be taken to view the colon mucosa (irrigation and aspiration of the bowel, etc.), which increases the duration of the procedure. Although constipation is associated with a lower cecal intubation rate, its effect on CIT is uncertain [6,16]. In our study, in 43 (70%) of 62 patients with constipation, the median CIT time was >5.17 min. Increased colon length, which is one of the causes of constipation, and insufficient bowel preparation caused by constipation may be the reason for the prolongation of CIT in these patients.

Longer CIT was detected in patients with a long CL and using additional analgesics. This situation seems logical. Additional analgesic administration due to increased pain is an indirect indication that the procedure is difficult. This may cause prolongation of CIT. Similar to our study, Lee et al. reported that shorter CL decreases CIT [21]. Excessive colon length may be associated with increased CL. Furthermore, loops, particularly in the sigmoid colon, may require more CL to complete the procedure. Both conditions contribute to the prolongs of the procedure.

Strengths and limitations

The strengths of this study are that it is prospective and includes more than one obesity index. However, it has some limitations. First of all, this is a single-center study. On the other hand, the fact that the procedures were performed by more than one experienced colonoscopist increases the study's generalizability. Undoubtedly, the most crucial factor affecting the CIT is the experience and skill of the colonoscopist. Because experienced colonoscopists conducted our study, the cecum intubation rate of 95% and the maximum CIT of less than 15 minutes, although this did not wholly exclude the experience-related factor affecting CIT, reduced it. Another limitation of the study is that the history of previous surgery was evaluated under a single heading. It has been reported that the history of gynecological surgery, in particular, prolongs the CIT [5]. Finally, BMI was divided into three groups. Subgroup analysis was not performed in obese patients according to the severity of obesity. Therefore, the CIT may be different in severely and morbidly obese (BMI >40). Additional studies are needed to understand whether the severity of obesity has an impact on CIT.

## Conclusions

In this study, factors affecting CIT were examined. Younger age, low WHtR, low BMI, increased CL, constipation, poor bowel preparation, and the use of extra analgesics were associated with longer CIT. When all factors were evaluated together, it was found that obesity measured only by WHtR (>0.5) and BMI (>30 kg/m2) are independent factors that reduce CIT.

Identifying factors that prolong the colonoscopy time can help endoscopists to be prepared, especially before the difficult colonoscopy, and may constitute a criterion for patient selection during the learning curve period.
